# Supplementary material for: Exploring the interplay between BMI, subjective body image perception, and health behaviors: A cross-sectional study
Source: PLoS One. 2025 Aug 14;20(8):e0328283. doi: 10.1371/journal.pone.0328283 (PMC12352643; doi:10.1371/journal.pone.0328283)
Supplement: Attachment — Submitted filename: Response to Reviewers.docx [file pone.0328283.s002.docx]

Ressponse to #1 Reviewer

Thank you for taking the time to provide valuable feedback and comments on manuscript. We have supplemented and revised the manuscript that you suggested. The additional and revised sections are listed below, with the modified parts highlighted in red italicized text and the manuscript locations marked in bold for easier find.

***Reviewer Comments:***

***Reviewer #1:***

1. Do not use the term “risk” in cross-sectional studies, as we cannot draw causal conclusions in this design due to the unclear temporal precedence. Please review all manuscripts accordingly.

*Thank you for your valuable comment. We agree that the term “risk” is not appropriate in the context of a cross-sectional study due to the lack of temporal precedence. Accordingly, we have carefully revised the manuscript to replace all instances of “risk” with more appropriate terms such as indicator, prevalence, concern, likelihood, etc.*

2. This is too long an introduction. Please shorten it into two or three paragraphs related to the main concepts, their relationship or other resulting health issues.

*Thank you for your valuable feedback. In response to your suggestion, we have significantly revised the introduction by reducing it from seven to three concise paragraphs. We focused on presenting the main concepts, their interrelationships, and the resulting health issues more clearly, while maintaining the logical flow and keeping the essential information.*

3. You had excluded the non-answers and answered them with “I don’t know”. Please provide more detail on these groups, such as missing tables or charts, to account for any bias or missing patterns in your work.

*Thank you for your helpful comment. As requested, we have clarified the treatment of missing responses and “I don’t know” answers in the manuscript. Specifically, the flow diagram (Figure 1) now includes a clear explanation regarding the handling of missing data and provides a detailed breakdown of excluded cases, including non-responses. In addition, participants who answered “I don’t know” were not excluded arbitrarily.*

4. What is the reason for excluding underweight people? Did you perform any sensitivity analysis with this group to ensure the validity of your results?

*Thank you for the insightful question. Individuals with an underweight BMI tend to exhibit distinct psychological and physiological characteristics compared to the general adult population. They are more likely to be associated with conditions such as eating disorders, nutritional deficiencies, or underlying illnesses. These individuals often demonstrate distorted body image perceptions, extreme dietary control, and exercise avoidance—factors that significantly differentiate their health behavior patterns from those of individuals within the normal to obese BMI range.*

*Based on these considerations, we excluded the participants who were underweight due to this subgroup heterogeneity, including underweight individuals could potentially confound the interpretation of the primary aim of this study, which is to examine the relationship between BMI–SBIP discrepancy and health behaviors in the general adult population.*

5. Please mention the cut-off point for obesity or overweight in the "Measurement" section in the method; the reference you cite in line 161 does not match the values given in line 185

*Thank you for your feedback. As different criteria were used to classify the degree of obesity, we revised the reference cited. Additionally, we added the specific cut-off values in line 198.*

***Line 198***

“The categorization of obesity levels based on BMI adhered to the standards set by Korean Society for the Study of Obesity BMI classification: Normal (18.5 kg/m^2^ ≤ BMI < 23 kg/m^2^), Overweight (23 kg/m^2^ ≤ BMI < 25 kg/m^2^), Obese (25 kg/m^2^ < BMI)”

6. Please clarify more about this definition, “Occupational status (white-collar, blue-collar, pink-collar, not classified),”

*Thank you for your specific suggestion. Based on your recommendation, we have explained occupational status in detail.*

**Line 175**

“The socioeconomic factors considered in this study included; Type of residence (Single-family house, Apartments, Others), Educational Level (Under Elementary school graduate, Middle-school graduate, High-school graduate, Over university graduation), Occupational status ~~(white-collar, blue-collar, pink-collar, not classified)~~, and Income quintile (low, lower middle, middle, upper middle, high).

Occupational status was classified into four categories based on the characteristics and typical work environments of the participants’ jobs. White-collar workers were identified as salaried professionals and office workers, such as managers and clerks, whose roles involve administrative or professional tasks not directly tied to physical production, while blue-collar workers were those engaged in manual labor, such as factory workers and laborers, typically in industrial or construction settings where they contribute directly to the production of goods. Pink-collar workers were defined as individuals in service-oriented or caregiving roles traditionally associated with women, such as nurses, teachers, and retail service workers, often requiring interpersonal and emotional skills. Occupations that could not be categorized due to insufficient or ambiguous information in the survey responses were labeled as not classified.”

7. Lines 189 and 190 are duplicated in the section on method. You have already mentioned this in section 2.1

*Thank you for the valuable feedback. Based on your comment, we have removed the duplicated content in line 190 to avoid redundancy.*

**Line 200**

“Participants who reported being ‘slightly obese were categorized as overweight, while those reporting as very obese were classified as obese. ~~This study excluded participants categorized as underweight or thin, focusing instead on those with normal weight, overweight, and obesity.~~ This classification was based on actual BMI measurements in conjunction with participants’ SBIP.”

8. The explanation of the weighting method in the calculation of total activity was misleading, please simplify.

*Thank you for your valuable comment. In response to your suggestion, we have simplified the explanation of the weighting method in the calculation of total activity.*

**Line 214**

“Participants were asked to report on their weekly engagement in each activity type, ~~physical activity for each category by answering the following three questions: (1) How many days were engaged in the activity? (2) How many hours did you spend on the activity per day? (3) How many additional minutes did you spend on the activity per day?~~ and the total minutes of MVPA per week were calculated using the equation: ~~The total number of minutes of MVPA per week was calculated using the equation:~~

Total minutes per week = (Hours per day × 60 + Additional minutes per day) × Number of days

~~Total minutes per week = (Hours per day × 60 × Number of days) + Additional minutes per day. In calculating the total~~To account for intensity differences, vigorous physical activity (VPA) minutes were weighted as twice the value of ~~from both work and leisure activities was given a twofold weight to~~ moderate physical activity (MPA) minutes before summing the total MVPA.”

9. In the statistical method, you should explain the independent and dependent variables in the multinomial logistic regression to provide more clarity.

*Thank you for your valuable suggestion. We have elaborated on the independent and dependent variables used in the multinomial logistic regression and specifically described them in detail.*

**Line 280**

“To examine the factors associated with discrepancies between SBIP and BMI,~~explore the relationships between variables such as SPBI and the degree of difference in BMI,~~ multinomial logistic regression analysis was performed. The dependent variable was the SBIP and BMI discrepancy group, categorized as Group A, Group B, and Group C. Independent variables included educational level, occupational status, individual income quintile, drinking and smoking status, perceived stress level, moderate-to-vigorous physical activity (MVPA), sleep duration, sedentary behavior, and health indicators such as fasting glucose, HbA1c, total cholesterol, HDL cholesterol, hypertension, and triglyceride levels.”

10. According to your results in Table 2, you have little data in these categories, especially in group c, so the chi-square test is not valid and appropriate to assess the transparency between groups in this case.

*Thank you for the valuable comment regarding the appropriateness of the chi-square test. As you pointed out, we were also concerned about the validity of the chi-square test given the relatively small number of participants in Group C. Therefore, prior to conducting the chi-square test, we carefully examined the expected cell frequencies for all categorical variables. As a result, all expected cell counts were greater than 5, satisfying the assumption required for the chi-square test. Thus, the application of the chi-square test was considered appropriate. We have added a clarification regarding this point in the revised manuscript for transparency as follows.*

**Line 275**

: “Prior to performing the Chi-square tests, we ensured that all expected cell counts met the minimum requirement of 5, thus satisfying the assumptions required for the chi-square analysis.”

11. The results reported in Table 3 are very vague as there is no clear methodology for variable selection, adjustments, co-linearity checking and interpretation of the multinomial models. Please revise the results and provide the necessary explanations for more clarity.

*Thank you for your insightful comment. Regarding your comment, we’ve added a sentence to describe the reference group as follows.*

**Line 283**

: “Group A was set as the reference category in the analysis.”

*Also, we described our variable selection criteria based on theoretical relevance and associations in univariate analysis as follows.*

**Line 288**

: “Independent variables were selected based on theoretical relevance and their associations in univariate analyses (*p* < 0.20). Multicollinearity was assessed using variance inflation factors (VIF), and all variables showed VIF values below 1.6, indicating no multicollinearity concerns.”

*Lastly, we clarified that no additional covariate adjustments were made as follows.*

**Line 293**

: No additional covariate adjustments were made in the models.

Ressponse to #2 Reviewer

Thank you for taking the time to provide valuable feedback and comments on manuscript. We have supplemented and revised the manuscript that you suggested. The additional and revised sections are listed below, with the modified parts highlighted in red italicized text and the manuscript locations marked in bold for easier find.

***Reviewer Comments:***

***Reviewer #2:***

**Abstract**

1. Abstract should be structured based on journal format

*Thank you for your feedback. We have changed format of abstract.*

2. Numerical values need to be added to the results.

*Thank you for your suggestion. We have added specific numerical values to the Results section to enhance clarity and detail.*

3. All abbreviations need to be defined (e.g OR, HDL, etc.)

*Thank you for your specific advice. We have added the full form of the abbreviations such as OR, HDL, and MVPA. Also, I have replaced “physical activity” with its abbreviation as follows.*

***Line 39***

“Group C had better High-Density Lipoprotein (HDL) cholesterol (59 mg/dL) and lower prevalence rates of hyperlipidemia (9.7%) and hypertension (5.5%) than Group B (hyperlipidemia: 11.6%; hypertension: 5.1%) and Group A (hyperlipidemia: 13.2%; hypertension: 7.6%). Moderate-to-Vigorous PA (MVPA) was significantly lower in Group C (97.88 min/week) than Group A (133.18 min/week; p < 0.05) and Group B (169.64 min/week; p < 0.05).”

***Line 46***

“Stress levels were significantly higher in those with lower BMI or higher SBIP (Odds Ratio [OR] = 1.93, *p* < 0.01). SBIP may have a stronger influence on health behaviors than BMI alone, particularly in shaping PA patterns.”

**Introduction**

4. This section is too long. unnecessary information needs to be removed or summarized.

*Thank you for your valuable feedback. In response to your suggestion, we have significantly revised the introduction by reducing it from seven to three concise paragraphs. We focused on presenting the main concepts, their interrelationships, and the resulting health issues more clearly, while maintaining the logical flow and keeping the essential information.*

5. line 67: define abbreviations

*Thank you for your feedback. We have added the full form of the abbreviation as requested.*

***Line 75***

“NBIP can reduce engagement in PA [11], contributing to poor management of risk factors such as elevated fasting glucose, Hemoglobin A1c (HbA1c), cholesterol levels, and blood pressure [12, 13]."

6. line 79: mention the exact number for proportion instead the word "significant"

*Thank you for your specific feedback. Based on your recommendation, we have added the exact number for proportion and reference.*

***Line 87***

“In 2016, approximately 26.5% of the adult population did not achieve the recommended PA levels, and the average time spent in SB was substantial [18].”

7. What is the gap of knowledge?

*Thank you for your insightful comment regarding the gap in current knowledge about our research. In response to your mention, we have made the additional sentence.*

***Line 117***

“While prior research has examined the impact of BMI and SBIP on health behaviors independently, this study uniquely integrated physical and psychological health factors to comprehensively understand of how these elements influence daily health behaviors and metabolic health [11, 32, 33]. The findings will be crucial for developing targeted interventions that address both mental and physical health, particularly in the context of increasing global obesity and sedentary lifestyles.”

**Methods**

8. One of the problems of the method section is that first there is a summary of methodology and then a comprehensive explanation of it. I suggest merging them and do not repeating them. For instance, the authors have explained about ABC categories and then in another section gave a complete explanation about categorization.

*Thank you for the valuable feedback. Based on your comment, we have removed the duplicated content and revised the manuscript to ensure each topic is described only once. Specifically, we have removed the explanation of the KNHANES survey from Section 2.1. ‘Study Participants’ and the categorization criteria for BMI from Section 2.3.1. ‘Anthropometric Measurements’*

9. Add proper reference for the first paragraph of 2.1.

*Thank you for pointing this out. Based on your comment, we have added reference that explained the second sentence in first paragraph.*

***Line 130***

“The KNHANES is a cross-sectional survey designed to evaluate the health and nutritional status of the Korean population [34].”

10. Why didn't the authors use previous cycles of KNHANES data?

*Thank you for your insightful question regarding the data that we used. We used the 8th KNHANES (2019–2021) data as it was the most recent cycle with complete data collection, given that 2024 data were not yet released. Additionally, KNHANES surveys vary across cycles in questionnaire items and variable definitions, so we selected a single cycle to ensure consistency in data collection methods and variable measurements. Furthermore, our study was designed as a cross-sectional study focused on the most recent period rather than long-term trends, our design did not require previous cycles.*

11. More details regarding physical examinations and data collections and measurements should be added (height, weight, etc.).

*Thank you for your specific suggestion. In response to your comment, we have added further details regarding the anthropometric measurements to provide a more comprehensive description.*

***Line 153***

“KNHANES gathers data via self-reported surveys and direct physical measurement using standardized instrumentation. Age and gender were obtained through~~using a~~ self-reported questionnaries~~survey~~, while anthropometric data were collected by trained medical staff during mobile health examination sessions. All physical measurements were taken twice, with a brief interval between trials, and recorded by a designated recorder to ensure consistency and accuracy.

Height was measured in centimeters using ~~the~~ a SECA 274 stadiometer (SECA, Germany) with participants standing upright in the Frankfurt plane position., Weight was measured in kilograms to the nearest 0.1 kg using ~~the~~ a GL-6000-20 scale (G-tech, South Korea), with participants wearing light clothing and no ~~and~~ shoes. ~~waist~~ Waist circumference (WC) was measured in centimeters at the midpoint between the lower margin of the rib cage and the iliac crest, after a normal exhalation, using W606PM tape measure (Lufkin, USA).”

12. Mention the inclusion and exclusion criteria and add a flow diagram for it.

*Thank you for your valuable feedback. We have added flow diagram about inclusion and exclusion of participants in method section. Accordingly, the original Figure 1 has been renumbered as Figure 2, Figure 2 as Figure 3, and Figure 3 as Figure 4.*

***Line 139***

**Fig 1.** Flow diagram.

13. line 141: SP < BMI? are you sure?

*Thank you for your careful comment. The corresponding content was removed in the process of revising the manuscript based on another reviewer’s feedback.*

***Line***

: “Participants were then divided into three groups based on the congruence between their BMI and SBIP categories:”

14. what do you mean by white, blue, and pink collar?

*Thank you for your specific suggestion. Based on your recommendation, we have explained about occupational status in detail.*

***Line 175***

“The socioeconomic factors considered in this study included; Type of residence (Single-family house, Apartments, Others), Educational Level (Under Elementary school graduate, Middle-school graduate, High-school graduate, Over university graduation), Occupational ~~status (white-collar, blue-collar, pink-collar, not classified)~~, and Income quintile (low, lower middle, middle, upper middle, high).

Occupational status was classified into four categories based on the characteristics and typical work environments of the participants’ jobs. White-collar workers were identified as salaried professionals and office workers, such as managers and clerks, whose roles involve administrative or professional tasks not directly tied to physical production, while blue-collar workers were those engaged in manual labor, such as factory workers and laborers, typically in industrial or construction settings where they contribute directly to the production of goods. Pink-collar workers were defined as individuals in service-oriented or caregiving roles traditionally associated with women, such as nurses, teachers, and retail service workers, often requiring interpersonal and emotional skills. Occupations that could not be categorized due to insufficient or ambiguous information in the survey responses were labeled as not classified.”

15. What is the definition of ex-smoker?

*Thank you for your insightful question. In our study, the definition of an ex-smoker is based on the Korea National Health and Nutrition Examination Survey (KNHANES) questionnaire, which categorizes smoking status into three groups: "currently smoking," "never smoked," and "smoked in the past but not currently smoking." Accordingly, we defined an ex-smoker as an individual who selected the response "smoked in the past but not currently smoking."*

16. Why didn't the author use alcohol volume rather than frequency of drinking?

*Thank you for pointing this out. We considered that the total alcohol consumption could vary significantly depending on both the frequency of drinking and the amount consumed per occasion. Additionally, we adopted the categorization ("Abstained in the last year," "Once a month," "2–4 times a month," "At least twice a week") because prior studies using the same KNHANES dataset have employed similar classifications. Accordingly, we have added a reference to support this approach.*

***Line 192***

“Drinking status was categorized into four groups (Abstained in the last year, Once a month, 2 – 4 times a month, At least twice a week) [35] and smoking status was categorized into three groups (Never smoked, Ex-smoker, Current smoker).”

**Results**

17. lines 301-321: there is no need to mention X2 values in the main text.

*Thank you for your comment. We have deleted all of the χ^2^ values that we mentioned in the lines 301 – 321.*

18. Table 2: Mention *p* values rather than X^2^ values.

*Thank you for your suggestion. In accordance with your comment, we have revised Table 2 to report p-values instead of Chi-square (X²) values.*

**Discussion**

19. Mention the cross-sectional design of the study as a limitation for causal relationship.

*Thank you for your valuable suggestion. We have added the following content to the end of the Discussion section.*

***Line 502***

“Furthermore, the cross-sectional design of this study presents a limitation in establishing a causal relationship between the discrepancy of BMI and SBIP and various health indicators. As a cross-sectional study analyzes data at a single point in time, it is challenging to determine temporal precedence or causality between variables. Therefore, future longitudinal studies are needed to address this limitation and provide clearer insights into the causal relationships among these factors.”
