# Supplementary material for: Exploring the interplay between BMI, subjective body image perception, and health behaviors: A cross-sectional study
Source: PLoS One. 2025 Aug 14;20(8):e0328283. doi: 10.1371/journal.pone.0328283 (PMC12352643; doi:10.1371/journal.pone.0328283)
Supplement: Attachment — Submitted filename: Response_to_Reviewers_auresp_2.docx [file pone.0328283.s003.docx]

Response to #1 Reviewer

Thank you for taking the time to provide valuable feedback and comments on manuscript. We have supplemented and revised the manuscript that you suggested. The additional and revised sections are listed below, with the modified parts highlighted in red italicized text and the manuscript locations marked in bold for easier find.

***Reviewer Comments:***

***Reviewer #1:***

1. Introduction was too long and should be shortened and summarized to make it readable for other researchers.

*Thank you for your valuable feedback. As requested, we have revised the Introduction section by reducing its length and summarizing the content to enhance clarity and readability for other researchers.*

2. If you report the results of multinomial logistic regression in the form of OR, you must not use the prevalence rate, but use the correct term such as odds or chance of disease.

*Thank you for your insightful comment. In response, we have carefully revised the manuscript to replace all instances where “prevalence” was used in the interpretation of multinomial logistic regression results. We now consistently use the appropriate terms such as “odds” or “chance of disease” to ensure accurate and proper interpretation of the findings.*

3. The exclusion of underweight people is not properly addressed! You simply deleted some sentences in this area of concern during the revision.

*Thank you for your comment. To address this concern, we have added a clear rationale for the exclusion of underweight individuals in section 2.3.5 Group. Specifically, we included the following explanation:*

***Line 204***

“Body image misperception and related behavioral outcomes, such as changes in PA, are commonly observed among individuals with normal, overweight, obese (Liechty, 2010). In contrast, underweight indviduals tend to exhibit consistently lower levels of PA and other distinct characteristics that differentiate them from these groups (Alwan et al., 2011; Eaton et al., 2008). Therefore, participants with a BMI below 18.5 kg/m2 and those who underweight or thin in their SBIP were excluded from this study.”

4. Ex-smoking is not yet clearly defined.

*Thank you for your pointing this out. To clarify the definition, we have added the following sentence to section 2.3.4. Lifestyle behaviors:*

“Ex-smokers were defined as individuals who had smoked in the past but were not smoking at the time of the survey.”

5. The method of physical activity assessing was not cited as a valid reference. Do you have a question or a valid measure for the method used?

*Thank you for your valuable suggestion. To address this issue, we have added the following clarification at the beginning of section 2.3.6. Health-related factors:*

“MVPA and SB were assessed using the Korean Version of Global Physical Activity Questionnaire, validated for Korean populations [39]. ST was measured using specific questions from the KNHANES”
